# Cyclostationary-Based Vital Signs Detection Using Microwave Radar at 2.5 GHz

**DOI:** 10.3390/s20123396

**Published:** 2020-06-16

**Authors:** Fatima Sekak, Kawtar Zerhouni, Fouzia Elbahhar, Madjid Haddad, Christophe Loyez, Kamel Haddadi

**Affiliations:** 1 CNRS, UMR 8520–IEMN groupe CSAM (Systems Circuits Microwave Applications), University of Lille, F-59000 Lille, France; christophe.loyez@univ-lille.fr (C.L.); kamel.haddadi@univ-lille.fr (K.H.); 2 Groupe LEOST (Electronic Wave and Signal Laboratory for Transport), University of Gustave Eiffel, F-59666 Villeneuve d’ Ascq, France; zerhouni.kawtar@gmail.com (K.Z.); fouzia.boukour@ifsttar.fr (F.E.); 3 Segula Engineering France, 92500 Rueil-Malmaison, France; madjid.haddad@segula.fr

**Keywords:** microwave radar, vital signs, respiration rate, hearth rate, cyclostationary, cyclic moment, cyclic cumulant

## Abstract

Non-contact detection and estimation of vital signs such as respiratory and cardiac frequencies is a powerful tool for surveillance applications. In particular, the continuous wave bio-radar has been widely investigated to determine the physiological parameters in a non-contact manner. Since the RF-reflected signal from the human body is corrupted by noise and random body movements, traditional Fourier analysis fails to detect the heart and breathing frequencies. In this effort, cyclostationary analysis has been used to improve the radar performance for non-invasive measurement of respiratory rate and heart rate. However, the preliminary works focus only on one frequency and do not include the impact of attenuation and random movement of the body in the analysis. Hence in this paper, we evaluate the impact of distance and noise on the cyclic features of the reflected signal. Furthermore, we explore the assessment of second order cyclostationary signal processing performance by developing the cyclic mean, the conjugate cyclic autocorrelation and the cyclic cumulant. In addition, the analysis is carried out using a reduced number of samples to reduce the response time. Implementation of the cyclostationary technique using a bi-static radar configuration at 2.5 GHz is shown as an example to demonstrate the proposed approach.

## 1. Introduction

Respiration rate (RR) and heart rate (HR) are considered the most important physiological parameters indicating the body’s functioning state. A variety of detection technologies of these vital signs have been proposed in the literature, including electro-cardiograms (ECG) [1], smartwatches [2], inertial sensors [3], ballistocardiograms (BCG) [4], infrared cameras [5], transducers sensors [6] and optical fibres [7,8]. The main disadvantage of these technologies is their physical contact with the patient, which in some cases are uncomfortable or impractical.

RF radars have been used in wireless sensors applications for decades. Compared to other technologies, wireless radars are interesting for their benefits of non-contact and non-invasive detection based on radio frequency (RF).

The advantages of non-contact vital signs detection by radar methods have been demonstrated in [9,10]. These vital signs are estimated from the acquired waveform that reflects the chest volume variation and displacement during the inspiration and expiration. The first use of radar systems for the detection of vital signs dates back to the 1975 s for measuring RR [11]. Since then, the interest in radars for the monitoring of breathing and heart rate has increased considerably. Most of the contactless concepts introduced are based on detecting minor movements of the chest, due to RR and HR. Both can be separated by dedicated signal processing algorithms. Among the different types of radar systems, we find continuous wave (CW) [12,13,14], six-port [15,16], frequency-modulated continuous wave (FMWC) [17,18] and ultra-wideband (UWB) [19,20,21] radars. The CW radar is better in comparison, due to higher measurement accuracy, less complex hardware architecture and simpler signal processing approaches.

In parallel, advanced detection signal processing methods have been reported in the literature. Linear and nonlinear methods, such as arc-tangent demodulation [22], complex demodulation [23], and adaptive DC calibration [24,25], have been proposed to directly demodulate the return signal phase to obtain vital-sign information. Other works have focused on different filtering techniques [26,27,28,29,30], varying the type and the order of the filter, as well as the cutoff frequencies of each filter, in order to isolate the cardiac signal from the respiratory signal. For example, in [30] the heartbeat signal was first separated from the respiration signal by a Butterworth bandpass filter (BPF) with pass-band 0.7–3 Hz, then the filtered signal was windowed, auto-correlated and the fast Fourier transform (FFT) was applied to obtain HR.

However, since the chest displacement caused by respiration is between 4 and 12 mm, and the chest displacement due to heartbeat alone ranges between 0.2 and 0.5 mm, the respiration signature is therefore much larger and stronger than the heartbeat component [31]. Heartbeat is more sensitive to the interference introduced by respiration harmonics and noise from remote sensing and the environment, which makes an accurate estimation difficult.

To solve the harmonic problem, the wavelet transform (CWT) [32] combined with either empirical mode decomposition (EMD) was introduced in [33]. In a follow-up paper [34], Ensemble Empirical Mode Decomposition (EEMD) was used. This technique consists of applying the EMD decomposition several times, then calculating the average of the modes. A curvelet transform was applied in [35] in order to remove the direct coupling wave and background clutters, then the signal was denoised using a singular value decomposition. Both the FFT and the Hilbert–Huang transform were applied in order to separate and extract HR and RR.

In the case of a low signal-to-noise ratio (SNR), it is difficult to obtain a precise heart rate in the presence of strong harmonics of RR [36]. To overcome these problems, the use of the cyclostationarity theory has been proposed. This theory is based on the estimation and the detection of vital signs from the signal contaminated by noise and random body motion to extract HR and RR. No any filtering, demodulation, or phase unwrapping, and insensitivity to SNR level are the advantages of this technique. It was first introduced for this purpose in [31,37]. In these reported works, the authors proposed using simulations tools for cyclostationarity to estimate the spectral correlation function (SCF) with a very large number of samples. The work [38], introduces the third-order cyclic cumulants (TOCC). However, the authors describe the higher-order cyclostationary for a zero mean value signal, this consideration makes the second cyclic cumulants and the third order cyclic cumulants both equivalent to their respective moments, while that is not true in this signal case. For this type of signal, the mean value is non-zero. Therefore, the second cyclic cumulants and the third cyclic cumulants are different from their respective moment and cyclic moment.

In this paper, we propose to develop the theoretical vital sign detection analysis based on the second order cyclostationary approach, using cyclic mean, cyclic conjugate autocorrelation functions and cyclic cumulant. To verify the detecting performances of the proposed method, a series of simulations and experiments considering different patients are carried out to confront the theory.

Moreover, we evaluate the impact of the numbers of samples and the noise while considering a small number of samples to reduce both the computational cost and the response time of the digital signal-processing block. The measurements are performed at the ISM frequency 2.5 GHz in a bi-static scenario using a CW radar, built up with a vector network analyzer interfaced with two horn antennas.

It has to be mentioned that the aim of this study is clearly oriented through the development and evaluation of the proposed algorithm. Therefore, the hardware part is based on a measurement laboratory equipment that offers the possibility to adapt the measurement configuration for the parametric analysis.

The remainder of this paper is organized as follows. Section 2 explains the model simulating vital signals. Section 3 presents the derivation of the received signal cyclic features. In Section 4 the impact of the stand-off distance, i.e., separation between the antennas and the patient, are investigated. Section 5 presents the experimental results of our proposed model and the extraction of the fundamental frequencies of heartbeat and breathing from the cyclic mean conjugate cyclic autocorrelation and cyclic cumulant. Furthermore, the performance of cyclostationary analysis is evaluated by mean of detection probability. Finally, Section 5 concludes this work.

## 2. Signal Model

A wireless bio-radar is composed of two blocks: the wireless transceiver and the signal processing block [36]. The transceiver is used to generate the transmitted CW and to convert the received signal to baseband. The signal processing block is used to estimate the heart and respiration frequencies. In fact, the respiration results in a quasi-periodic extension of the chest, that is in the range of millimeters.

The heartbeat induces a quasi-periodic motion that is superimposed to the respiration, and is in the submillimeter range. The CW transmitted signal, that is generated by the local oscillator signal, can be written as follows:

(1) $$T\left(t\right)=\cos\left(2\pi ft+\varphi (t)\right).$$

Let us consider that the target is located at a distance *d*. Furthermore, let us define the time-varying chest displacement as $x_{HR}\left(t\right)$ and the random body motion as $x_{I}\left(t\right)$, then the propagating electromagnetic wave travels the distance 2d(t), where $d\left(t\right)=d+x_{HR}\left(t\right)+x_{I}\left(t\right)$. The chest displacement varies between 4 mm and 12 mm due to RR, while it ranges between 0.2 mm and 0.5 mm due to HR. The relation between the phase variation Δθ and the chest displacements $x_{HR}\left(t\right)$ is shown in (2):

(2) $$\Delta \theta =\frac{4\pi x_{HR}\left(t\right)}{\lambda },$$

where λ is the free-space wavelength of the transmitted signal and $\lambda =\frac{c}{f}$ (*c* represents the speed of light and f RF frequency of operation). Hence the reflected signal from the body can be expressed as follows:

(3) $$R\left(t\right)=A_{HR}\cos\left[2\pi ft-\frac{4\pi d}{\lambda }-\frac{4\pi x_{HR}\left(t\right)}{\lambda }-\frac{4\pi x_{I}\left(t\right)}{\lambda }\right.\left.+\varphi \left(t-\frac{2d}{c}\right)\right]+N\left(t\right)$$

where $x_{I}\left(t\right)$ can be modeled as a two-dimensional (2D) quasi-periodic motion considered as two independent 1D motions [31]. Each 1D motion is modeled as a stationary random process with a uniform distribution in a specified direction.

The signal of interest is $x_{HR}\left(t\right)=x_{r}+x_{h}$. It denotes the displacement generated by respiration and heartbeat. The heart signal $x_{h}$ (resp. the respiration signal $x_{r}$) is nearly periodic, with a time-varying period. At rest, the heartbeat rate varies between 50 and 90 beats per minute [36]; this corresponds to a frequency between 0.83 and 1.5 Hz, respectively. On the other hand, the resting respiration rate varies between 9 and 24 breaths per minute; this corresponds to a frequency between 0.15 and 0.4 Hz [39]. Both can be further developed as follows:

(4) $$x_{h}\left(t\right)=a_{h}\cos\left(2\pi f_{h}t+\theta_{h}\left(t\right)+\theta_{h0}\right)$$

(5) $$x_{r}\left(t\right)=a_{r}\cos\left(2\pi f_{r}t+\theta_{r}\left(t\right)+\theta_{r0}\right),$$

where $a_{h}\left(t\right)=\Lambda_{h}+\alpha_{h}\left(t\right)$ and $a_{r}\left(t\right)=\Lambda_{r}+\alpha_{r}\left(t\right)$. The amplitude and phase variations $\alpha_{h}\left(t\right)$ and $\theta_{h}\left(t\right)$ (resp. $\alpha_{r}\left(t\right)$ and $\theta_{r}\left(t\right)$) are zero mean random processes. They model the phenomenon of heart rate variability (resp. respiration rate variability) [36]. However, in this paper, we consider a constant approximation of these values, by carefully choosing the sensing interval T, so that:

(6) $$x_{h}\left(t\right)=a_{h}\cos\left(2\pi f_{h}t\right),\;t\in \left[(k-1)T,kT\right]$$

(7) $$x_{r}\left(t\right)=a_{r}\cos\left(2\pi f_{r}t\right),\;t\in \left[(k-1)T,kT\right].$$

In order to simplify the notation, the amplitudes $a_{hk}$ and $a_{rk}$ will be denoted $a_{h}$ and $a_{r}$ respectively.

Once the reflected signal is mixed with the local oscillator, the baseband quadrature signals can be expressed as follows:

(8) $$I\left(t\right)=A_{HR}\cos\left[\frac{4\pi d}{\lambda }+A_{r}\cos\left(2\pi f_{r}t\right)\right.\left.+A_{h}\cos\left(2\pi f_{h}t\right)+\frac{4\pi x_{I}\left(t\right)}{\lambda }+\Delta \varphi \left(t\right)\right]+N_{I}\left(t\right)$$

(9) $$Q\left(t\right)=A_{HR}\sin\left[\frac{4\pi d}{\lambda }+A_{r}\cos\left(2\pi f_{r}t\right)\right.\left.+A_{h}\cos\left(2\pi f_{h}t\right)+\frac{4\pi x_{I}\left(t\right)}{\lambda }+\Delta \varphi \left(t\right)\right]+N_{Q}\left(t\right),$$

where

(10) $$\Delta \varphi \left(t\right)=\varphi \left(t\right)-\varphi \left(t-\frac{2\pi }{c}\right)$$

(11) $$A_{r}=a_{r}\frac{4\pi }{\lambda }\;and\;A_{h}=a_{h}\frac{4\pi }{\lambda }.$$

The baseband signal can be simplified to its complex form:

(12) $$Bb\left(t\right)=A.M\left(t\right)exp\left[jA_{r}cos\left(2\pi f_{r}t\right)\right].exp\left[jA_{h}cos\left(2\pi f_{h}t\right)\right]+Z\left(t\right),$$

where

(13) $$A=A_{HR}exp\left(j\frac{4\pi d}{\lambda }\right)$$

(14) $$M\left(t\right)=exp\left[j(\frac{4\pi x_{I}\left(t\right)}{\lambda }+\Delta \varphi \left(t\right))\right]$$

and $Z\left(t\right)=N_{I}\left(t\right)+jN_{Q}\left(t\right)$ is the complex noise. According to Jacobi Anger expansion:

(15) $$e^{jzcos\alpha }=\sum_{q=-\infty }^{\infty }j^{q}J_{q}\left(z\right)e^{jq\alpha },$$

where $J_{q}$ represents the *q*-th-order Bessel function of the first kind, the heart and respiration signals in (12) can be expanded as Bessel series. Therefore, the baseband signal can be simplified into the following equation:

(16) $$Bb\left(t\right)=AM\left(t\right)\sum_{q=-\infty }^{\infty }\sum_{l=-\infty }^{\infty }j^{q+l}J_{q}\left(A_{r}\right)J_{l}\left(A_{h}\right)exp\left[j2\pi (qf_{r}+lf_{h})t\right]+Z\left(t\right).$$

It is clear from (16) that the random body motion, the distance separating the radar and the target as well as the operating frequency are all parameters that impact the RR and the HR estimation and detection. Furthermore, as the reflected signal is corrupted by additive and multiplicative noise, the periodicity of the signal of interest can be hidden. However, these periodicities can be extracted using cyclostationarity signal processing. In fact, in severe noise and interference environments, cyclostationarity-based algorithms have been shown to outperform conventional techniques [40]. In this context, the next section presents the cyclic statistics of the received signal (16).

## 3. Cyclostationarity of Vital Signs

A signal is a wide sense almost-cyclostationary of order *m* if its statistics of order *m*, (moments and cumulants) are almost periodic functions of time [41]. This built-in periodicity can be extracted and analysed, using Fourier analysis, leading to the definition of temporal cyclic moments (TCMF) and temporal cyclic cumulants (TCCF). We focus on extracting the cyclostationarity moments of order 1 and 2 of the received baseband signal and the second cyclic cumulant (15), to detect RR and HR.

The phase noise and random body motion in (16) result in a random amplitude that is considered as a multiplicative noise and modeled by M(t).

The first order of the algorithm which is the cyclic mean is a Fourier transform in terms of the cyclic frequencies, in contrast to the standard Fourier analysis which as a function of the frequency. Those cyclic frequencies are is a combination of the respiratory rate and the heart rate, which differ from one order to another. Then, we proposed the second order of the approach to make more difference. The cyclic mean of the baseband received signal can be defined as follows [42]:

(17) $$CM_{y}\left(\alpha \right)=\lim_{T\to \infty }\frac{1}{T}\int_{\frac{-T}{2}}^{\frac{T}{2}}E[AM\left(t\right)\sum_{q=-\infty }^{\infty }\sum_{l=-\infty }^{\infty }j^{q+l}J_{q}\left(A_{r}\right)J_{l}\left(A_{h}\right)exp\left[j2\pi \left(qf_{r}+lf_{h}\right)t]+Z\left(t\right)\right]exp\left(-j2\pi \alpha t\right)dt.$$

The noises Z(t) and M(t) are uncorrelated and stationary [3]. Furthermore, using the Dirac delta identity

(18) $$lim_{T\to \infty }\frac{1}{T}\int_{\frac{-T}{2}}^{\frac{-T}{2}}exp\left(\pm j2\pi ft\right)\;dt=\delta \left(f\right)$$

the cyclic mean in (16) can be derived as:

(19) $$CM_{y}\left(\alpha \right)=A\mu_{M}\sum_{q=-\infty }^{\infty }\sum_{l=-\infty }^{\infty }j^{q+l}J_{q}\left(A_{r}\right)J_{l}\left(A_{h}\right)\delta \left(\alpha -\left(qf_{r}+lf_{h}\right)\right)+\mu_{Z}\delta \left(\alpha \right),$$

where $\mu_{M}$ and $\mu_{Z}$ represent the mean of the multiplicative noise M(t) and the additive noise Z(t) respectively.

If $\mu_{M}\neq 0$, then the baseband received signal is first order almost-cyclostationary, with a set of cyclic frequencies $A_{\alpha }=\{\pm (qf_{r}+lf_{h}),0\}$. As it be noticed, the cyclic frequencies are a combination of heart and respiration rates. Hence, in this case, the vital signs can be extracted from the first-order cyclic moment (the cyclic mean). However, if $\mu_{M}=0$, then defining the cyclic autocorrelation of y(t) is of a great use.

The conjugate cyclic autocorrelation function is defined as follows [43]:

(20) $$R_{yy}\left(\alpha ,\tau \right)=\lim_{T\to \infty }\frac{1}{T}\int_{\frac{-T}{2}}^{\frac{T}{2}}y\left(t\right)y^{*}\left(t+\tau \right)exp\left(-j2\pi \alpha t\right)\;dt.$$

Replacing y(t) with the signal model in (16), we can write:

(21) $$\begin{array}{cc} R_{yy}\left(\alpha ,\tau \right) & =A^{2}R_{M}\left(\tau \right)\lim_{T\to \infty }\frac{1}{T}\int_{\frac{-T}{2}}^{\frac{T}{2}}\sum_{q=-\infty }^{\infty }\sum_{l=-\infty }^{\infty }\sum_{q^{\prime }=-\infty }^{\infty }\sum_{l^{\prime }=-\infty }^{\infty }J_{q}\left(A_{r}\right)J_{l}\left(A_{h}\right)J_{q^{\prime }}\left(A_{r}\right)J_{l^{\prime }}\left(A_{h}\right)\\ \; & exp\left[j2\pi (\left(q-q^{\prime }\right)f_{r}+\left(l-l^{\prime }\right)f_{h})t\right]exp\left[-j2\pi (q^{\prime }f_{r}+l^{\prime }f_{h})\tau \right]exp\left(-j2\pi \alpha t\right)\;dt+R_{Z}\left(\tau \right),b \end{array}$$

where $R_{M}\left(\tau \right)$ and $R_{Z}\left(\tau \right)$ are the autocorrelation functions of the stationary noises M(t) and Z(t). Using the aforementioned Dirac delta identity, the cyclic autocorrelation function of the baseband signal can simplified to:

(22) $$\begin{array}{c} R_{yy}\left(\alpha ,\tau \right)=A^{2}R_{M}\left(\tau \right)\sum_{q=-\infty }^{\infty }\sum_{l=-\infty }^{\infty }\sum_{q^{\prime }=-\infty }^{\infty }\sum_{l^{\prime }=-\infty }^{\infty }J_{q}\left(A_{r}\right)J_{l}\left(A_{h}\right)J_{q^{\prime }}\left(A_{r}\right)J_{l^{\prime }}\left(A_{h}\right)exp\left[-j2\pi (q^{\prime }f_{r}+l^{\prime }f_{h})\tau \right]\\ \delta \left(\alpha -\left(\left(q^{\prime }-q\right)f_{r}+\left(l^{\prime }-l\right)f_{h}\right)\right)+R_{Z}\left(\tau \right) \end{array}$$

Provided that $R_{M}\left(\tau \right)\neq 0$, the conjugate cyclic autocorrelation of y(t) function is defined for the set of cyclic frequencies $A_{\alpha }=\left\{\pm (\left(q^{\prime }-q\right)f_{r}+\left(l^{\prime }-l\right)f_{h}),0\right\}$.

The *n*-th order cyclic temporal cumulant function (cyclic cumulant) is a Fourier coefficient of the cyclic temporal cumulant function. The cyclic cumulants are pure sine-wave amplitudes, and they can be computed by combining cyclic moment amplitudes. The cyclic cumulants can be given from the cyclic moment conversion, that is given by the combination of products of lower-order cyclic temporal moment functions:

(23) $$C_{x}^{\nu }{\left[\tau \right]}_{m,p}=\sum_{D_{m}}\left[{\left(-1\right)}^{d-1}\left(d-1\right)!\sum_{\alpha^{⊺}1=\nu }\prod_{i=1}^{d}R_{x}^{\alpha_{i}}{\left[\tau_{bi}\right]}_{m_{i},p_{i}}\right],$$

where ν is the pure sine wave of the lower order cyclic frequencies and α is the impure sine-wave of order m. The vector of cycle frequencies α is the vector of cyclic temporal moment cycle frequencies, and they must sum up to the cyclic-cumulant cycle frequency ν.

In (23), the sum is over distinct partitions of the index set {1, …, *m*}, referred to as $D_{m}$, *d* is the number of elements in a partition,1⩽d⩽m. The set of indexes belonging to a partition is denoted as ${\left\{b_{i}\right\}}_{i=1}^{d}$.

Table 1 presents the set of partitions for the order m=2. The number of possible partitions increases with the order, and is given by the Bell’ s number.

The conversion of cyclic moments into cyclic cumulants has the following interpretation. In fact, in the literature, the set of cyclic frequencies defined from the cyclic moments is referred to as “impure cyclic frequencies”, as it can be the result of a combination of lower order cyclic frequencies. Whereas, the cyclic frequencies defined from the cyclic cumulants are known as the “pure cyclic frequencies”, as they characterize the m-th order additive sine-waves only. In other words, cyclic cumulants isolate the cyclic feature of the m-th order from products of lower orders. They represent the part of the m-th order moment sine wave that is independent of all lower-order moment sine waves. Hence, all possible products of lower-order pure sine-waves can be subtracted from the m-th order cyclic moment sine waves to obtain the cyclic cumulant of order m [43].

**Figure 1:**
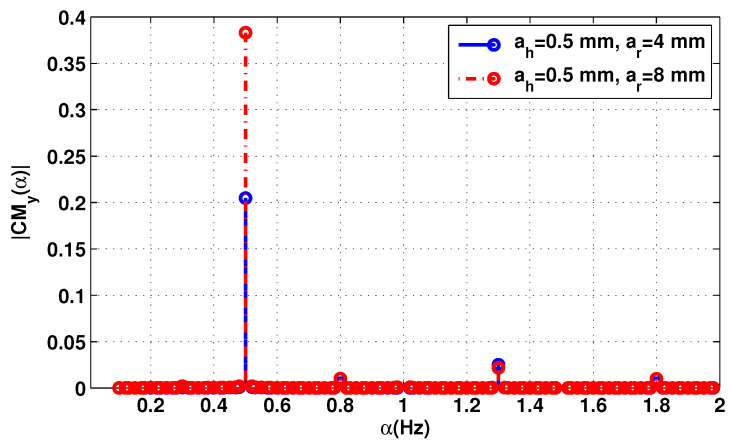
First cyclic moment of the baseband signal $B_{b}\left(t\right)$ without additive noise, f=2.5 GHz.

**Figure 2:**
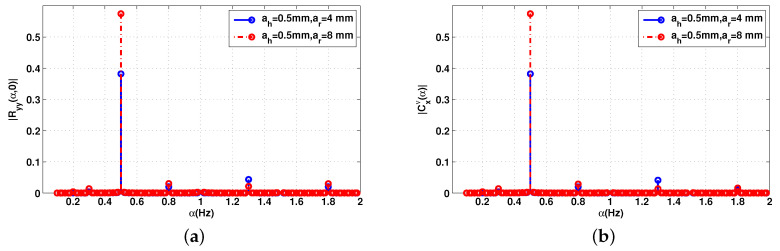
Second-order of cyclic statistics of the baseband signal $B_{b}\left(t\right)$ without additive noise, f=2.5 GHz: (**a**) conjugate cyclic autocorrelation; (**b**) cyclic cumulant second order.

Figure 1 and Figure 2 show the cyclic mean, conjugate cyclic autocorrelation and second cyclic cumulant of the received signal at the frequency 2.5 GHz. In order to focus only on the cyclic features of the vital signs, the signal is generated without neither the additive nor the multiplicative noise, and the attenuation $A_{HR}$ has not been considered. In this example, the distance separation between the antennas and the patient is set to 60 cm.

As can be noticed, all the cyclic statistics contain the heart and respiration frequencies, $f_{h}$ = 1.3 Hz and $f_{r}$ = 0.5 Hz. The frequencies combination $f_{h}-f_{r}$ = 0.8 Hz and respiration harmonic $2f_{r}$ = 1 Hz represent the second and third peaks of both cyclic statistics. The respiration signal is usually the component of the lowest frequency in cyclic components and is at the same time the strongest component of the signal because of the chest movement due to respiration $a_{r}$ = 4 mm and $a_{r}$ = 8 mm. It is therefore more important than the heartbeat $a_{h}$ = 0.5 mm. This makes it easier to extract the breathing frequency, while precise heart rate detection is the main challenge. In the first configuration, where $a_{r}$ = 4 mm these harmonics are less significant than the heart peak. However, in the second configuration ($a_{r}$ = 8 mm), the $2f_{r}$ is greater than the heart peak.

**Figure 3:**
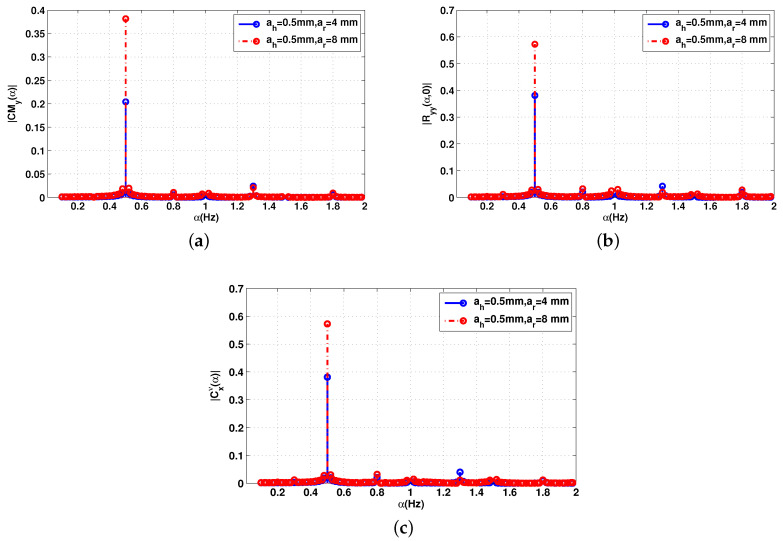
Cyclic statistics of the baseband signal $B_{b}\left(t\right)$ without noise, f=2.5 GHz. (**a**) Cyclic mean; (**b**) conjugate cyclic autocorrelation; (**c**) cyclic cumulant second order.

The impact of the number of samples is considered in Figure 3. The cyclic features of the received signal for the 2.5 GHz frequency and distance d=60 cm, with a small number of samples (Ns=601) are presented. All frequencies of interest are present. However, the cyclic autocorrelation estimation is corrupted with the noise. This means that the cyclostationarity process requires a high number of samples to accurately estimate the cyclic features.

Our goal in this paper is to evaluate the impact of the numbers of samples and distance between the radar and the antenna, on cyclic features of the received signal, and how they affect the detection of vital signs.

## 4. Influence of Distance on Cyclostationary Detection

In our framework, we extend the study to include the impact of noise and distance on cyclic features detection. To this end, we rely on the following definitions.

The attenuation $A_{HR}$ for the complex-valued reflected signal is [28]:

(24) $$A_{HR}=\sqrt{\frac{P_{Tx}G_{T}G_{R}\sigma L_{h}\lambda^{2}}{{\left(4\;\pi \right)}^{3}d^{4}}},$$

where $P_{Tx}$ is the transmitted power, $G_{T}$ and $G_{R}$ are transmitter and receiver antenna gains. σ is the RCS, $L_{h}$ is the reflection loss of the heart.

The residual phase noise power from the vital signs can be given by [2]:

(25) $$N_{\Delta \varphi_{h}}=\frac{P_{Tx}G_{T}G_{R}G_{Rx}\sigma L_{h}}{2\pi f^{2}d^{2}}S_{\varphi }\left(1\right)ln\left(\frac{f_{max}}{f_{min}}\right),$$

where $G_{Rx}$ is the receiver gain. $S_{\varphi }\left(1\right)$ is the phase noise at 1 Hz. As can be seen from (25), the residual phase is inversely proportional to both the operating frequency as well as the distance. This power is used to generate the residual phase noise $\Delta \varphi \left(t\right)$ in Equation (14).

Finally, the thermal noise is expressed as follows [2]:

(26) $$N_{Thermal}=8G_{Rx}kTBN_{f},$$

where *k* is Boltzman’ s constant, *T* the absolute temperature, *B* is the bandwidth. $N_{f}$ is the noise figure. Unlike phase noise, thermal noise is independent from distance and frequency. It means that the noise is fixed regardless of the attenuation of the reflected signal. The thermal noise power is used to generate the additive complex noise Z(t).

Furthermore, from the attenuation Equation (24) we can derive the maximum distance at which detection of vital signs is possible:

(27) $$d_{max}={\left[\frac{P_{Tx}G_{T}G_{R}G_{Rx}\sigma_{h}L_{h}\lambda^{2}}{{\left(4\;\pi \right)}^{3}A_{HR_{min}}^{2}}\right]}^{\frac{1}{4}}.$$

Equation (27) shows that the detection distance is closely related to the transmitted power, and antenna parameters, as the frequency test. These variables are constant and transceiver-dependent, while the target RCS σ and loss *L*, are related to the human body.

Using these definitions, the signal (16) is generated for the investigated frequencies. The bio-radar parameters are summarized in Table 2.

As the heart rate is in the range of 0.83–1.5Hz, the minimum sampling frequency allowing reconstruction of cyclostationarity should be at least twice the maximum target frequency $f_{s}\geq 2\;max\left(f_{h}\right)$, in this case $f_{s}\geq 4\;Hz$. The sampling rate is then chosen to minimize the computational cost, $f_{s}$ = 100 Hz. Hence, the signal is generated with a sampling time of $T_{s}=0.01\;s$, and for a total observation time T=60s, which leads to $N_{s}=6001$ samples. In [31], the results are obtained using 131,072 samples. One of our main goals is to require cyclic features by reducing the measurement time and the numbers of samples, hence we carry out our frequency/distance analysis using small sample sizes.

**Figure 4:**
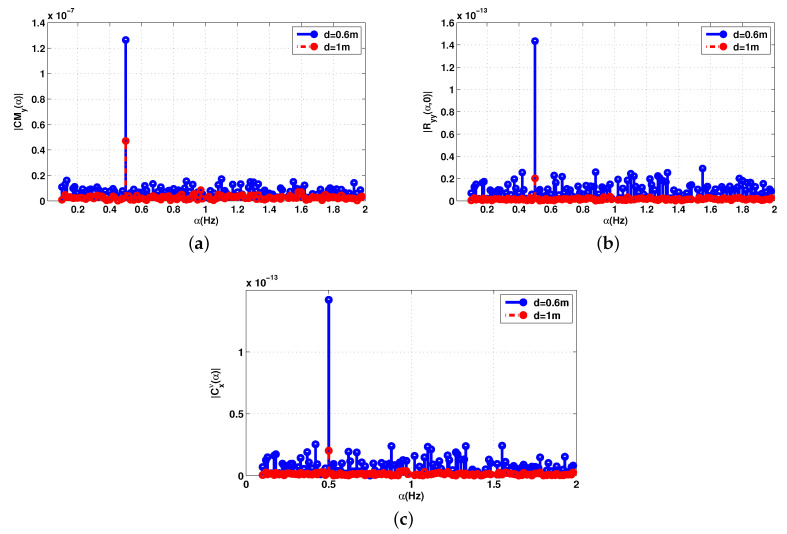
Cyclic statistics of the baseband signal $B_{b}\left(t\right)$ with additive noise, SNR = 0 dB. (**a**) Cyclic mean; (**b**) conjugate cyclic autocorrelation; (**c**) cyclic cumulant second order.

Figure 4 represents the cyclic features of the received signal in (15) for the 2.5 GHz frequency and two distinct distances d=60 cm and d=1 m and the number of samples was set at 6001. In these examples, HR and amplitude were $f_{h}$ = 1.3 Hz and $a_{r}$ = 0.5 mm, respectively, while RR and amplitude were $f_{r}$ = 0.5 Hz and $a_{r}$ = 4 mm, respectively. As can be seen in Figure 4, all frequencies of interest are present and the number of samples is sufficient to reconstruct the cyclic spectrum. However, for *d* = 1 m, HR is submerged in noise. It should be noted that increasing the number of samples would lead to a better estimation of cyclic features at the expense of higher computational costs, which leads to a longer processing time.

**Figure 5:**
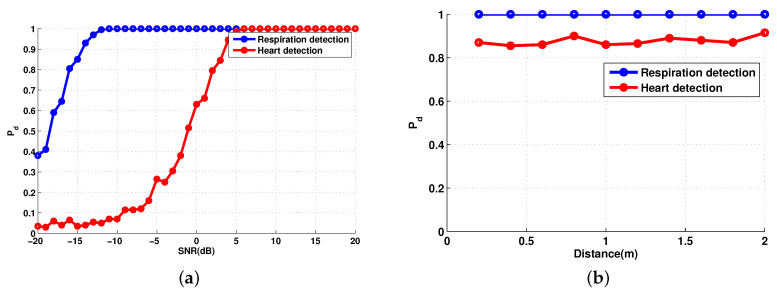
Probability of detection of vital signs: (**a**) as a function of the signal-to-noise ratio (SNR) and d=60 cm; (**b**) as a function of the distance and SNR = 0 dB.

To further assess the impact of the distance on detection of vital signs, we computed the probability of detection as a function of distance and a function of SNR, in order to detect the presence of both heart and respiration rates. We performed the detection as a function of the distance (Figure 5a) and a function of the SNR (Figure 5b), using sampling time value $T_{s}$ = 0.01 ($f_{s}$ = 100 Hz), which leads to $N_{s}$ = 6001 samples. Therefore, for simulation, we tested if the estimate values using the cyclostationary algorithm $f_{h}^{\prime }$ and $f_{r}^{\prime }$ of HR and RR respectively, are the same values set for the transmitted signal $f_{h}$ and $f_{r}$.

Thus we simulate both probabilities $P_{d}$ = $P(f_{r}^{\prime }=f_{r})$ and $P_{d}$ = P ($f_{h}^{\prime }$ = $f_{h}$). The blue curve corresponds to RR, while the red one corresponds to HR. The probability of detection of the respiration rate is $P_{d}$ = 1 for any distance and any level of SNR because the respiratory signal is the strongest component in the reflected signal. On one hand, the heart rate detection is around $P_{d}$ = 0.85 for each distance values. On the other hand, the probability of detection of the heart rate is increasing with the SNR.

The simulation results demonstrate that the cyclostationary theory is a powerful tool to extract the cyclic frequencies from noisy non-stationary signals independent of the SNR level, having taking into consideration the body movements, the attenuation and the additive and the multiplicative noises.

## 5. Experimental Validation

A radar system generally consists of a transmitter, antennas, a receiver and signal processing hardware and/or software. The transmitter creates a waveform. A directional antenna concentrates the beam in the direction of the target. The receiver converts the signal from the transmission frequency to an intermediate frequency or to a baseband. Signal processing is used to reject clutter and out-of-band noise while passing the desired signal, and to derive information from the signal.

**Figure 6:**
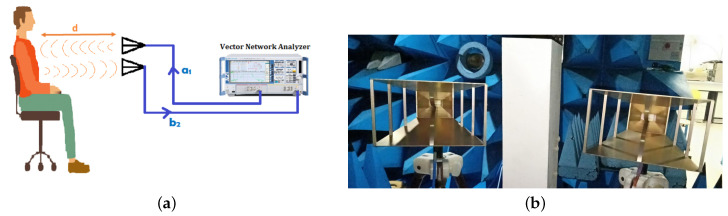
Measurement configuration considering a vector network analyser (VNA) and two horn antennas.(**a**) System design operating at f=2.5 GHz; (**b**) the double ridge guide horn antenna 0.7–18 GHz.

The radar system shown in Figure 6 is built up with a reference measurement system, i.e., vector network analyser (VNA 24 from Rohde and Schwarz R&S, 0.7–24 GHz) interfaced with two horn antennas (AH Systems SAS-571 Double Ridge Horn Antenna 700 MHz–18 GHz ) through stable coaxial cables.

The VNA provides continuous waves at the desired frequency 2.5 GHz with an RF output power set to 0 dBm. The antenna is directed to the patient’s thorax which is placed at a distance of 60 cm from the patient. The RF signal radiated by the antenna ($a_{1}$) is connected to the port 1 of the VNA, which is reflected from the person’s chest and received by the second antenna ($b_{2}$) connected to the port 2 of the VNA, is driven to the VNA where the phase-shift arg(S21) and the amplitude variations of the complex transmission coefficient $|S_{21}|=\frac{b_{2}}{a_{1}}$ are determined (Figure 6). Hence, it contains information about the heartbeat and the breathing frequencies. Therefore, the most important feature is that the RF signal goes through clothing with minimal reflection and has higher reflection on the skin interface.

According to (2), the phase-shift variation, for the test frequency 2.5 GHz (λ = 120 mm) is 1.2${}^{\circ }$ for a displacement of 0.2 mm, and 3${}^{\circ }$ for 0.5 mm. Many parameters are accessible to be set by using a VNA, such as the frequency, the number of measurement points, the sweep time, and hence, the sampling rate.

It is necessary to reduce the transmitted power in order to reduce the radiated energy to which the patient is exposed during the measurements. Various organizations and countries have developed exposure standards for radio frequency energy [44]. These standards recommend safe exposure levels for the general public and workers. Then, it is important to note that the selected frequency belongs to the Industrial Scientific Band (ISM) and the transmitted power does not exceed the limits specified by several organizations, such as the institute of Electrical and Electronics Engineers (IEEE), and the Federal Communications Commission (FCC) have issued recommendations for human exposure to RF electromagnetic fields.

Table 3 shows the features of The SAS-571 double ridge guide horn antenna, and the Table 4 shows the parameters defined for the VNA.

For this quantitative measurement campaign, we consider a reference commercial kit of Libelium MySignals [45] for HR and RR. MySignals is a development platform for medical devices and eHealth applications. It can measure more than 15 different biometric parameters with several sensors. For our case, we use the two sensors ECG and AIRFLOW that are fixed on the patient and at each test we note the value of the HR in beats per minute (BPM) and RR. The data gathered by MySignals is encrypted and sent to the developer’s private account at the Libelium Cloud. Table 5 presents general features of MySignals.

**Figure 7:**
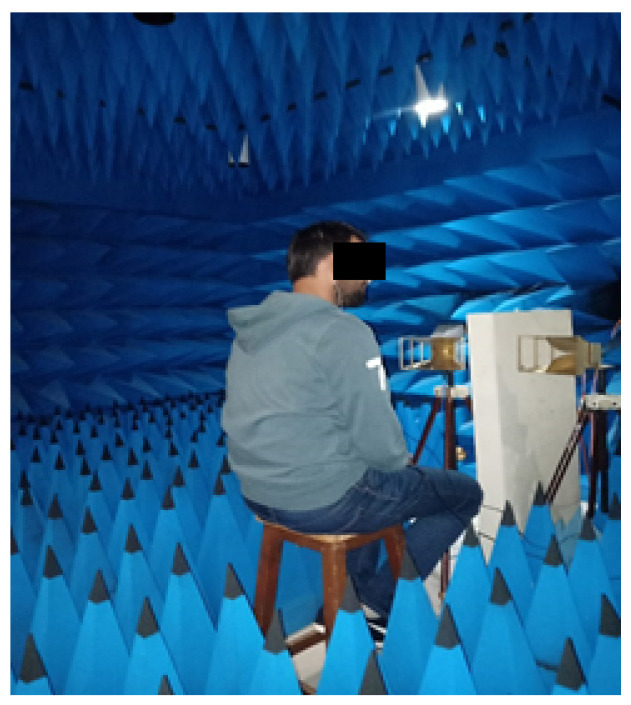
Measurement system operating at 2.5 GHz.

In order to validate and assess the performance of the proposed algorithm and avoid disturbing the measurements by any other type of signal coming from the environment or multi-path or noise, we carried out a series of tests on two clothed voluntary subjects in an anechoic chamber (Figure 7) (patient 1 is a man aged 30, and the patient 2 is a man aged 45).

For the purpose of determining the effect of the distance between the antenna and the subject detected chest, the distance is set in two values d=60 cm and d=1 m, the test frequency is set at 2.5 GHz, while keeping other parameters constant and using the same experimental setup as described in Table 5. The subject sits on a chair for the duration of the measure, which is 114.63 s. The antennas are mounted on a fixed platform, which are directed and parallel to the subject’s chest.

**Figure 8:**
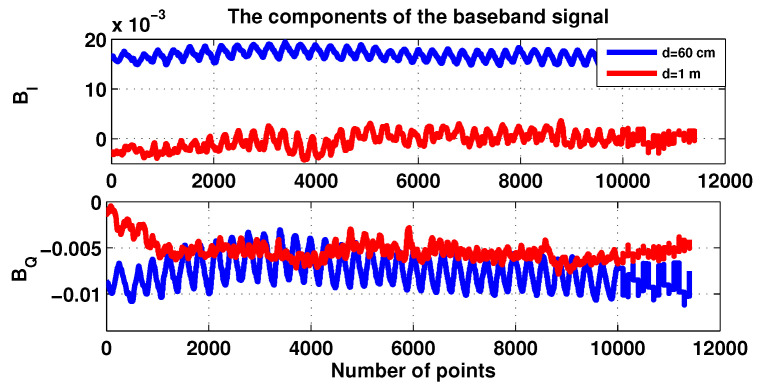
The components I and Q for patient 1.

Figure 8 shows the components of real part $B_{I}$ and the imaginary part $B_{Q}$ of the baseband signal plots for the two distances 60 cm and 1 m.

**Figure 9:**
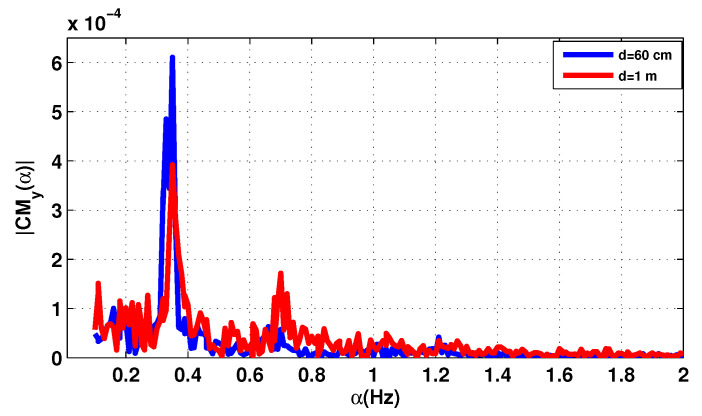
The cyclic mean for the patient 1.

**Figure 10:**
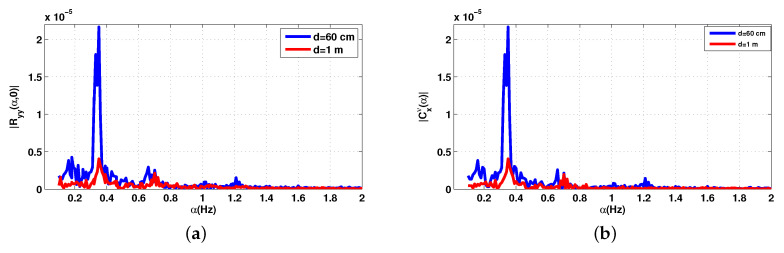
Cyclic statistics order 2 of the received signal of the first patient, f=2.5 GHz. (**a**) Conjugate cyclic autocorrelation; (**b**) cyclic cumulant 2.

The cyclic features of the first and second order of the received signal with the measuring system of Figure 6 are shown in Figure 9 and Figure 10 for the two distances d=60 cm and d=1 m.

For the two distances, the desired breathing frequency $f_{r}$ and the heartbeat frequency $f_{h}$, the second and the third harmonics of breathing $2f_{r}$, $3f_{r}$ have been observed, since the breathing signal is usually the more important component compared to that of the heartbeat. It is easier to extract the signal due to breathing while the precise detection of the heart rate is done using a band pass filter, its frequency band chosen is based on the interval of the heartbeat which is 0.83–1.5 Hz.

The maximum amplitude frequency at 0.3489 Hz for d=60 cm is attributed to the RR, and 0.3 Hz for d=1 m. However, the peaks corresponding to the harmonics of the breathing frequency can sometimes be very close to the frequency of the heartbeat, which makes the evaluation of this heartbeat frequency difficult. In this case, the level of the heartbeat signal is low and can be also corrupted with noise, which make the distinction difficult. The HR is attributed to the frequency corresponds to the maximum amplitude, then the HR for d=60 cm and d=1 m are 1.204 Hz (61.44 bpm) and 1.02 Hz (61.2 bpm) respectively.

The reference of HR and RR provided by MySignals are shown together in Table 6 with the values detected by the cyclostationarity algorithm. It can be seen that the detected frequencies of RR and HR match with the reference values.

As the respiration signal is a relatively strong signal as compared to the heart signal it is not affected as much as the heart signal by the free space losses, clutter and noise from the environment that increase with the distance separation between the antennas and the human body.

**Figure 11:**
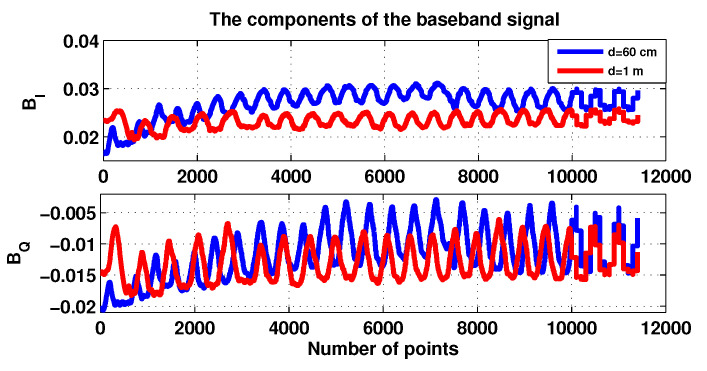
The components I and Q for patient 2.

**Figure 12:**
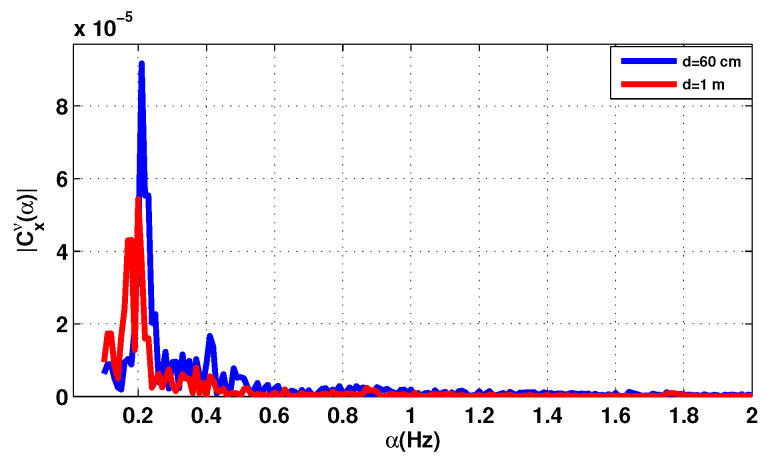
The cyclic features first order for patient 2.

**Figure 13:**
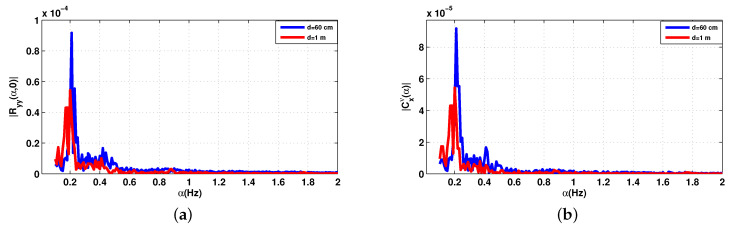
The cyclic statistics second order for the patient 2, f=2.5 GHz. (**a**) Conjugate cyclic autocorrelation; (**b**) cyclic cumulant 2.

The second measurement is related to the second patient aged 45. Figure 11 shows the components of the baseband signal for the second subject. Figure 12 and Figure 13 show the cyclic features first and second order of the received signal Figure 7. The distance between the subject under test and the antenna is sat at two different values; 60 cm then 1 m.

The RR is detectable with considerable accuracy for any distance, because it is less affected by noise as well than the HR. The results show that increasing the distance between the antenna and the chest decreases the accuracy of the vital signs detection. However, the level of the heartbeat signal is more obvious than before.

The heartbeat signal was obtained by using the band-pass filter in the heartbeat band [0.83, 1.5] Hz which correspond to [50, 90]beats per minute. The maximum amplitude in this interval corresponds to the heart rate of the person under test.

With the reference device, the values of the HR are close to the values detected by the cyclostationary algorithm for the two distances shown in Table 7.

The shape of the measured signal differs slightly from one patient to another. For patient 1, the raw results of I and Q, we identified random movements of the body. The second order of the cyclic statistics for d=1 m were very attenuated compared to d=60 cm. However, the cyclic features of the patient 2 are more obvious than before for both distance 60 cm and 1 m compared to the patient 1. For the patient 2, the components I and Q of the baseband signal are clear and periodic, so the cyclic features will be better than those of the patient 1 which explain the differences in figures of both patients. The peaks corresponding to the harmonics of RR can sometimes be very close to HR which can make it difficult to evaluate this frequency. The level of the parasitic noise, physiological or electromagnetic origin, linked to the environment infects the detection of the HR which is a small component of the signal comparing to the RR.

**Figure 14:**
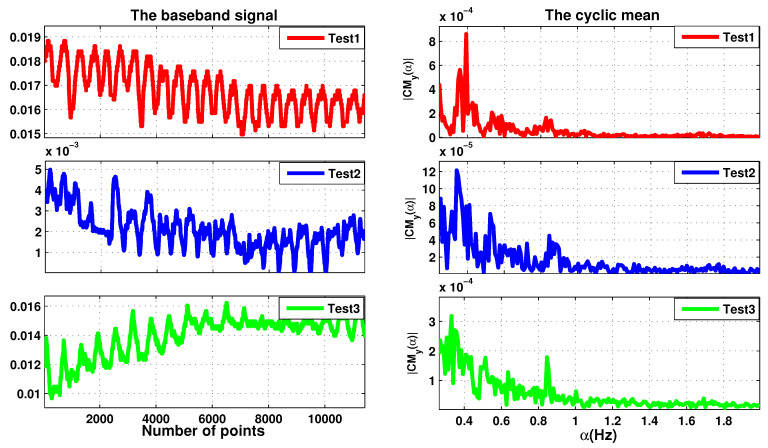
The baseband signal measured in different positions of the person-under-test and their cyclic means.

The second set of measurements is performed at 2.5 GHz. Three realistic measurement configurations are considered with different positions of the person-under-test: (i) the subject sits at 60 cm in front of the radar (TEST 1), (ii) same as (i) with body movement (TEST 2), (iii) the antenna is directed towards the back of the subject under test (TEST 3). From the measured signals, the cyclic means are computed and presented in Figure 14.

Table 8 summarizes the data for the measurements taken with the radar placed in front of the person under test without movement, and with movement and on the subject’s back. The amplitude of breathing is higher for measurements in front of the subject, which could be linked to the physiology and the anatomical position of the lungs in the thoracic cage. Through the back, the lungs are located deeper, and the signal is further attenuated by human tissue. This could make it easier to measure the heartbeat, however, the level of the heartbeat signal is low and difficult to measure due to the noise level and the body movement.

These results using the cyclostationary signal processing give a guideline for the parameters choice. Specially, the operating frequency should be chosen carefully if detection for longer distances is of interest. In fact, although cyclostationary analysis is robust against additive Gaussian noise, the nature of vital signs imposes stringent requirements. As can be seen from the above analysis, cyclic features vary in higher frequencies. An interesting finding in this paper is the behaviour of the 2nd order cyclic features related to RR and HR. The proposed algorithm provided an excellent performance. It is verified that the proposed method based on cyclic mean, conjugate cyclic autocorrelation and cyclic cumulant order 2 can detect the RR and HR. Combining the three functions we find the same results as the contact reference.

## 6. Conclusions

In this paper, we investigate the use of cyclostationary signal processing for vital signs detection. More precisely, we assess the impact of noise and distance on the cyclic features first order and second order of the reflected signal from the human body. A thorough presentation of the reflected signal affected by random body motion, residual phase noise and thermal noise is first presented. Afterwards, the cyclic features of order 1 and 2 are derived. Our analysis is based on the use of a limited number of samples, which reveals that the cyclostationary detection performance is indeed dependent on the target distance and SNR values. Hence, in a future work, a thorough analysis of higher order cyclic statistics will be carried out, and a combination of different orders and higher frequencies will be investigated for a better detection and estimation of vital signs.

## Figures and Tables

**Table 1:** Partitions, example for m=2.

$D_{m}$	{1,2}	{1} {2}
*d*	1	2
$b_{i}$	$b_{1}=\left\{1,2\right\}$	$b_{1}=\left\{1\right\}$
		$b_{2}=\left\{2\right\}$

**Table 2:** Simulation Parameters.

System Specification	Parameter
Operating frequency	[2.4, 5, 60] GHz
Distance	[0:25:300] cm
Receiver Gain $G_{Rx}$	10 dB
Antenna Gain $G_{T}=G_{R}$	0 dBi
Output power $P_{Tx}$	0 dB
Radar cross section (RCS) σ	6.8 × 10${}^{-3}$ m${}^{2}$
Heart Reflectivity *L*	−60 dB
Noise figure $N_{f}$	6 dB
Phase Noise at 1Hz $S_{\varphi }\left(1\right)$	58 dB/Hz

**Table 3:** Features of the horn antenna.

Block	Specifications
Frequency range	700 MHz–18 GHz
Gain (dBi)	1.4 to 15
Maximum Power	300 Watts

**Table 4:** Specifications of the measurement system.

Block	Specifications
Acquisition time	114.63 s
Distance	60 cm
Transmitted Power	0 dBm
Number of points	11,423
IFBW	100 Hz

**Table 5:** General features of MySignals.

Features	MySignals SW
Number of sensors	16
Sensor Readings	From any sensor (16) to one interface
Architecture	Libelium IoT Core
Radios on board	BLE(Bluetooth Low Energy), WiFi

**Table 6:** Comparison of the results for the patient 1.

	MySignals	Cyclostationarity
RR d=60 cm	0.35 Hz	0.3489 Hz
HR d=60 cm	1.204 Hz	1.15 Hz
RR d=1 m	0.35 Hz	0.3 Hz
HR d=1 m	1.04 Hz	1.1 Hz

**Table 7:** Comparison of the results for the patient 2.

	MySignals	Cyclostationarity
RR d=60 cm	0.22 Hz	0.21 Hz
HR d=60 cm	0.9 Hz	0.89 Hz
RR d=1 m	0.22 Hz	0.2 Hz
HR d=1 m	0.9 Hz	0.89 Hz

**Table 8:** Comparison of the results of respiration rate (RR) and heart rate (HR) with the cyclostationarity approach and the reference.

		Test1	Test2	Test3
RR	Cyclostationarity	0.3926 Hz	0.3402 Hz	0.3315 Hz
	MySignals	0.39 Hz	0.33 Hz	0.33 Hz
HR	Cyclostationarity	0.8288 Hz	0.8462 Hz	0.8462
	MySignals	0.83 Hz	0.85 Hz	0.85 Hz

## Abbreviations

The following abbreviations are used in this manuscript:

RR	Respiration Rate
HR	Heart Rate
VNA	Vector Network Analyzers
CW	Continuous Wave
SNR	Signal Noise Ratio
BPM	Beat Per Minute
FFT	Fast Fourier Transform
